# Time to screen: rationale and roadmap for HFpEF screening in individuals with obesity

**DOI:** 10.1007/s10741-025-10540-z

**Published:** 2025-06-23

**Authors:** Anouk Achten, Lukas Peeters, Geert Verkoulen, Jerremy Weerts, Christian Knackstedt, Evert-Jan Boerma, Vanessa van Empel, Sandra Sanders-van Wijk

**Affiliations:** 1 Department of Cardiology, Maastricht University Medical Centre, Cardiovascular Research Institute Maastricht (CARIM), PO Box 5800, 6202 AZ Maastricht, The Netherlands; 2 Department of Cardiology, Zuyderland Medical Center, Heerlen, The Netherlands; 3 Department of Surgery, Zuyderland Medical Center, Heerlen, The Netherlands

**Keywords:** Screening, Obesity, Heart failure with preserved ejection fraction, Diagnosis

## Abstract

Obesity is a major risk factor for heart failure with preserved ejection fraction (HFpEF) and is associated with a distinct pathophysiological phenotype. Individuals with obesity develop HFpEF on average a decade earlier than individuals without obesity. Despite this, systematic screening for HFpEF in individuals with obesity is not currently recommended in clinical guidelines. This review discusses the rationale for screening individuals with obesity for HFpEF, highlighting the rising prevalence of obesity-related HFpEF, its earlier onset, and its often under-recognized clinical presentation. We describe the specific pathophysiological mechanisms linking obesity to HFpEF, including inflammation, adipose tissue distribution, and hemodynamic alterations. Furthermore, we review the limitations of current diagnostic approaches in this population, including the interpretation of natriuretic peptides, echocardiographic challenges, and indexing pitfalls. Current and emerging screening tools (e.g., HFpEF-ABA score) are critically appraised, with a proposal for a stepwise screening and diagnostic pathway tailored to individuals with obesity. Given the high burden and early onset of HFpEF in people with obesity, screening strategies may enable earlier detection and timely intervention. Prospective studies are needed to determine the prevalence of (early) HFpEF in populations with obesity and to evaluate the effectiveness of structured screening approaches in clinical practice.

**Graphical Abstract:**

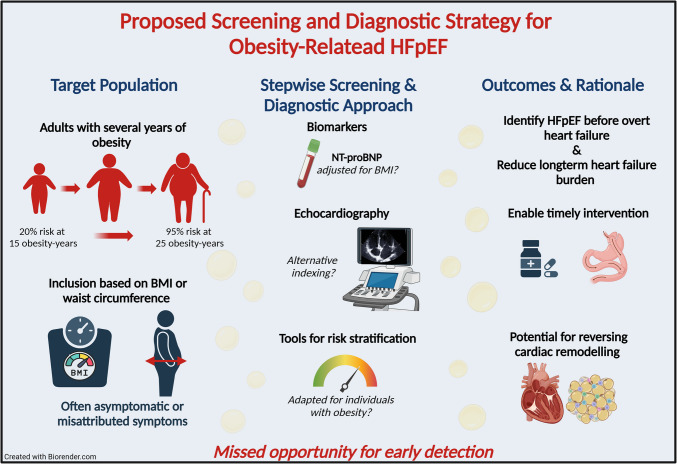

## Introduction

Obesity has become a global epidemic, impacting more than 650 million adults, and is a significant risk factor for various common and life-threatening conditions, including heart failure (HF) [1–3], particularly HF with preserved ejection fraction (HFpEF). The incidence of HF rises with age and excess weight, and obesity is notably prevalent in HFpEF patients, affecting approximately 48% of cases [4–6]. Moreover, the risk of developing obesity-related cardiac structural changes increases with the number of years a person has obesity—from 20% after 15 years to 95% after 25 years [7]. As a result, HFpEF in patients with obesity often develops at a younger age, typically a decade earlier than other HFpEF subtypes [7]. Consequently, individuals with obesity face a life-threatening disease at a relatively young age.

Early recognition and timely treatment of risk factors are crucial to prevent the development of HFpEF and, thereby, reduce significant disease burden for the patient as well as healthcare expenses. Since obesity is an important and highly treatable risk factor, the relationship between HFpEF and obesity has received more attention over the years. Nevertheless, most studies focus on the obese population already diagnosed with HFpEF. As a result, the prevalence of HFpEF in a general population with obesity is still relatively unknown. Moreover, the question of whether and how systematic screening for HFpEF in asymptomatic or mildly symptomatic individuals with obesity should be implemented remains unresolved. We will discuss in this review the value of screening as well as the bottlenecks and unmet needs in screening for HFpEF in individuals with obesity.

## Pathophysiology of obesity-related HFpEF

Research has shown that patients with obesity-related HFpEF exhibit specific pathophysiological characteristics—such as increased right ventricular dysfunction, pericardial restraint, and more pronounced cardiac remodeling—and thus, obesity-related HFpEF is considered a specific phenotype called “obesity-HFpEF” [4, 8]. There is growing interest in elucidating the pathophysiological mechanisms by which obesity contributes to the development of HFpEF. Evidence suggests that, beyond the total amount of fat, the distribution of adipose tissue plays a crucial role in obesity-related disorders [9, 10], likely due to both metabolic and local mechanical effects [11]. Visceral adipose tissue is more commonly linked to metabolic dysfunction than subcutaneous fat, as adipocytes in visceral adipose tissue secrete adipokines that trigger monocyte and macrophage infiltration into visceral adipose tissue, contributing to both local and systemic inflammation [12–14]. Additionally, visceral adipose tissue accumulation is associated with excessive triglyceride deposition in the myocardium, as well as adipose tissue buildup in the epicardial and pericardial regions [15, 16]. HFpEF patients with obesity have an increased amount of epicardial adipose tissue, which also functions as a metabolically active reservoir that affects the myocardium by releasing inflammatory cytokines [9, 17, 18]. These cytokines contribute to low-grade inflammation in the body and negatively affect endothelial function [19], leading to microvascular dysfunction and fibrotic remodeling. While inflammation is known to be present in HFpEF overall, it has also been reported that patients with the obesity HFpEF phenotype exhibit higher levels of systemic inflammation compared to HFpEF patients without obesity [8].

Additional to the metabolic effects, there are local mechanical effects. Epicardial fat exerts a detrimental mechanical effect, particularly during diastole, by occupying space and potentially contributing to elevated intracardiac pressures in HFpEF patients with obesity [4]. As a result, the amount of epicardial adipose tissue accumulation is associated with an adverse prognosis in HF patients [20]. It is, however, still unknown which mediators contribute the most and at which stage of the disease process. Likely, an interplay between the metabolic activity of the adipocytes, their abundance, and their locations all impacts the systemic consequences and the cardiac response. Mediators in the early stages of the disease are particularly interesting for future targeted interventions.

## Screening for heart failure in individuals with obesity

Screening for HF in individuals with obesity is not routinely performed, while screening for comorbidities in patients with established HF is recommended in current guidelines. Specifically, the current European Society of Cardiology (ESC) HF guidelines recommend screening for comorbidities such as diabetes, sleep apnea, and iron deficiency in patients diagnosed with HFpEF [21]. Similarly, the ESC cardiovascular risk management guidelines advise screening for diabetes in individuals with obesity and recommend assessing natriuretic peptides to detect HF in patients with diabetes [22]. However, no specific guidance is provided regarding HF screening in individuals suffering from obesity, but without diabetes, leaving a gap in current recommendations. The American Heart Association introduced a cardio-kidney-metabolic framework that emphasizes the importance of screening for HF in individuals with cardiometabolic risk profiles, including those with obesity, diabetes, and chronic kidney disease [23]. Although their approach incorporates kidney function and focuses broadly on multimorbidity, it supports the principle of proactive identification and intervention in at-risk individuals, in alignment with the screening rationale discussed here. Recently, a screening score for HFpEF has been developed, prior to employing natriuretic peptides or echocardiography, which includes obesity as a risk factor. The HFpEF-ABA score incorporates three clinical factors—age, body mass index (BMI), and a history of atrial fibrillation—to efficiently assess the likelihood of underlying HFpEF and identify patients who should be referred for an echocardiogram [24]. This suggests that screening for HF could be integrated into the care of patients with obesity; however, how effective this screening approach is in a population with obesity remains uncertain.

### Signs and symptoms for heart failure

Screening for HF is often performed in the presence of signs and symptoms of HF. A preliminary small study indicates that 31% of a population with obesity (BMI ≥ 30 kg/m^2^) and dyspnea symptoms meets the HFpEF criteria according to echocardiogram and invasive right heart catheterization [25]. Nevertheless, it is still unknown how many patients with obesity already exhibit signs of HFpEF without being symptomatic (early stage or “early-HFpEF”). Relying on signs and symptoms for HF might not be the best approach, as symptoms are often attributed to obesity itself, potentially leading to underdiagnosis. Common HF symptoms, such as shortness of breath, reduced exercise capacity, and fluid retention—manifesting as weight gain and ankle swelling—are also frequently observed in individuals with obesity due to excess body weight [26–28]. This overlap makes it challenging to distinguish whether symptoms are driven by HFpEF or are solely a consequence of obesity, complicating the diagnostic process. Furthermore, patients with obesity often have difficulty engaging in physical activity due to musculoskeletal issues, such as joint pain and osteoarthritis, which cause discomfort and reduced mobility. As a result, they may not notice exertional breathlessness, leading to underreported symptoms and potential delays in diagnosis [29].

#### Screening with natriuretic peptides

Natriuretic peptides, such as B-type natriuretic peptide (BNP) and N-terminal pro B-type natriuretic peptide (NT-proBNP), are crucial biomarkers for diagnosing HF and are incorporated into the ESC guidelines for diagnosing HFpEF [21]. It has been demonstrated that circulating levels of natriuretic peptides are lower in individuals with obesity, regardless of the presence of acute decompensated HF. However, these levels remain above the threshold used to rule out chronic HF [8, 30]. Several studies have shown that a proportion of HFpEF patients present with natriuretic peptide levels below the diagnostic threshold in whom the BMI is often increased [31, 32]. More recently, a multicenter study confirmed that in patients with unexplained dyspnea and invasively confirmed HFpEF, the sensitivity of the ESC-recommended NT-proBNP cutoff of 125 pg/mL decreased from 77 to 67% among those with BMI > 35 kg/m^2^. Of note, 48–63% of these patients were on baseline diuretics, which may also influence natriuretic peptide levels [33]. To conclude, it is well known that natriuretic peptides are influenced by factors such as obesity, kidney function, age, and sex [34] in patients without HF. These findings support that natriuretic peptide thresholds may require adjustment in obese HFpEF, though prospective screening studies in diuretic-naïve populations are needed to establish reliable diagnostic cutoffs without influence of diuretics.

#### Screening with echocardiography

Measuring natriuretic peptides alone is insufficient to diagnose HFpEF in the absence of acute decompensated HF; therefore, an echocardiogram is the next step in the diagnostic process. Two established scoring systems for HFpEF diagnosis [35, 36] incorporate various echocardiographic parameters, including E/e′ to assess diastolic dysfunction, left atrial volume index, left ventricular mass index, and estimated right ventricular pressure. The HFA-PEFF (Heart Failure Association Pretest Probability of Heart Failure with Preserved Ejection Fraction) and H2FPEF (heavy, hypertension, atrial fibrillation, pulmonary hypertension, elder, and filling pressures) scores assist in diagnosing HFpEF by quantifying its risk through a numerical scoring system. However, both scores classify a significant proportion of suspected HFpEF patients as having an intermediate likelihood, for whom further diagnostic testing is recommended [37, 38]. Thus, the choice of scoring system determines which patients are referred for additional testing or classified as having HFpEF, potentially leading to different clinical decisions. Atrial fibrillation and BMI were the main contributors to the discrepancy between the scores as both are key components of the H2FPEF score, while atrial fibrillation increases the diagnostic threshold for HFpEF in the HFA-PEFF score by increasing the NT-proBNP cutoff [35–37]. Therefore, it is advised that both scores are used next to each other [37]. A diagnosis of HFpEF is confirmed if either score is high, whereas HFpEF is excluded if both scores are low. The actual diagnostic performance of these scores in a specific population with obesity is unknown. In cases of an intermediate score, additional assessment with stress exercise echocardiography or invasive hemodynamic measurements is recommended. However, invasive right heart catheterization may carry a higher risk of complications in patients with severe obesity [39]. Also, performing echocardiography in individuals with obesity presents its challenges. Excess adipose tissue can attenuate ultrasound waves resulting in poor image quality [40]. Nevertheless, when the technician is given adequate time to apply necessary technical adjustments, the majority of key parameters can still be accurately assessed [27]. Additionally, adjusting echocardiographic parameters for body surface area (BSA) might be unreliable in individuals with obesity. Since BSA is largely determined by fat mass, this correction may lead to an overestimation of parameters indexed to BSA—such as left atrial volume—potentially diminishing the observed abnormalities [41]. Previous studies have therefore suggested indexation of left atrial volume to height^2^ and left ventricular mass to height^2.7^ [42, 43].

### HFpEF likelihood scoring in individuals with obesity

It is indeed possible to consider using another scoring system in individuals with obesity, though it still needs to be validated. Recently, a new scoring system was developed using machine learning, identifying BMI, estimated glomerular filtration rate, left ventricular mass index, and the left atrial to left ventricular volume ratio as the strongest predictors of HFpEF in obese patients [44]. This new scoring system was shown to outperform the H2FPEF score but was not compared to the HFA-PEFF score. Moreover, the control group in this study consisted of asymptomatic patients, raising uncertainty about how the score will perform in real-life clinical scenarios where HFpEF is suspected based on symptoms or other clinical, laboratory, or imaging findings. Additionally, the median BMI of the control group was 26.99, considerably lower than that of the HFpEF patients included in the study, which may impact the usage of this score to screen patients with obesity for HFpEF.

### Proposed screening algorithm

Screening for HFpEF in individuals with obesity holds substantial promise, yet several barriers must be addressed before it can be broadly implemented. An initial step may involve population-level risk stratification using clinical tools such as the HFpEF-ABA score, in combination with natriuretic peptide assessment. However, it is crucial to assess whether BMI is the most appropriate criterion or if waist circumference would be a better measure for selecting patients for screening, as it correlates more strongly with visceral and epicardial adipose tissue [45]. Individuals identified as high-risk can then undergo a targeted diagnostic work-up, including tailored echocardiographic evaluation that accounts for obesity-related limitations in image quality and indexing. Where diagnostic uncertainty persists, adjunctive testing—such as exercise stress echocardiography or invasive hemodynamic assessment—may be warranted. Nevertheless, prospective studies are warranted to investigate the prevalence of HFpEF in a population with obesity. These studies can also evaluate the current scoring systems and potentially lead to the development of a new score.

## Treatment

Following the identification of Obesity-HFpEF through screening, initiation of treatment with sodium-glucose cotransporter-2 (SGLT2) inhibitors is appropriate. This is particularly relevant given that obesity often coexists with dysglycaemia and hypertension. SGLT2 inhibitors not only confer clinical benefits in HF symptoms and outcomes but also modestly reduce body weight, glycated hemoglobin, and blood pressure [46]. SGLT2 inhibitors demonstrate consistent efficacy in reducing the primary composite outcome of worsening heart failure or cardiovascular death across the entire BMI spectrum, as shown in the DELIVER trial [46]. Treatment with SGLT2 inhibitors was also associated with an improvement in symptoms, with a greater magnitude of benefit observed in patients with higher BMI. Moreover, SGLT2 inhibitors consistently induce a modest but significantly greater reduction in body weight among individuals with higher BMI compared to those with lower BMI [46].

Obesity represents a significant yet modifiable comorbidity in patients with HFpEF [20]. Notably, individuals with obesity-related HFpEF exhibit a worse prognosis compared to other phenotypes, even after adjustment for comorbid conditions [47]. Early identification of HFpEF in individuals with obesity, coupled with targeted interventions promoting effective weight loss, holds particular promise—not only for preventing progression to overt heart failure, but also for improving clinical outcomes and facilitating reverse cardiac remodeling. Metabolic bariatric surgery (MBS) has proven to be an effective treatment for obesity, providing sustained benefits and higher rates of weight loss than conventional approaches [48, 49]. Research in obese individuals without pre-existing HF has indicated that MBS significantly lowers the risk of developing HF compared to lifestyle interventions alone [50]. Additionally, observational studies suggest that in patients with established HF, MBS is associated with improved quality of life and a reduction in HF-related hospitalizations [51, 52]. However, these studies have not focused specifically on HFpEF. Also, the effects of MBS on parameters such as left ventricular mass, chamber size, and overall cardiac function have been inconsistently reported. It remains uncertain whether the improved outcomes following MBS are primarily driven by weight loss or also by direct improvements in underlying cardiac function or structure [53, 54].

Pharmacological therapy for obesity has increasingly been recognized as a key strategy in recent years. In 2023, a randomized study involving 529 patients with obesity (BMI > 30) and HFpEF demonstrated that treatment with semaglutide, a glucagon-like peptide-1 (GLP-1) agonist, led to an improvement in quality of life after 1 year [55]. However, it is well established that weight loss achieved with MBS significantly exceeds that of GLP-1 agonists, with average reductions of ~ 35% versus ~ 15%, respectively [49, 55]. In combination with glucose-dependent insulinotropic polypeptide (GIP), GLP-1 agonists can achieve weight reductions of up to 25% [56]. Moreover, tirzepatide, combination therapy of GLP-1 and GIP, showed to reduce left ventricular mass and epicardial adipose tissue [57]. Nonetheless, discontinuing GLP-1 agonists is associated with weight regain in approximately 70% of patients [58]. In contrast, MBS combined with lifestyle intervention is capable of maintaining weight loss after 5 years in over 85% of patients [49]. Nonetheless, the indication for MBS is typically limited to patients under 65 years of age, while the average age of a typical HFpEF patient is around 75 years old. However, screening for HFpEF in a population suffering from obesity could allow for earlier diagnosis.

The extent to which early diagnosis and timely intervention, particularly through effective weight reduction, can reverse cardiac remodeling in individuals with obesity-HFpEF remains insufficiently understood. It is also unknown whether a threshold exists beyond which myocardial structural changes become irreversible, despite metabolic improvement. Importantly, recent evidence suggests that GLP-1 receptor agonists—alone or in combination with GIP—as well as MBS may exert cardioprotective effects that extend beyond weight loss. These interventions have been shown to reduce systemic inflammation, improve endothelial function, and attenuate myocardial fibrosis, potentially translating into improved clinical outcomes in HFpEF patients regardless of weight trajectory [56, 59–61].

## Conclusion

Systematic screening for HFpEF in individuals with obesity represents a clinically actionable opportunity to shift from late-stage recognition to proactive disease interception. Given the rising prevalence of obesity and its strong association with earlier onset and worse prognosis of HFpEF, timely identification of at-risk individuals is essential, especially with the availability of effective pharmacological and surgical therapies that may target underlying pathophysiologic mechanisms. A stepwise screening and diagnostic approach is proposed, beginning with clinical risk stratification and natriuretic peptide assessment, followed by echocardiographic evaluation tailored to the technical and interpretive challenges posed by obesity. Key research priorities include establishing obesity-specific thresholds for biomarkers, optimizing imaging indexation, and validating tailored diagnostic pathways. Prospective studies are now warranted to define the prevalence of (early) HFpEF in asymptomatic individuals with obesity, evaluate existing and emerging screening tools, and determine whether early diagnosis and intervention can halt or even reverse disease progression. Addressing these knowledge gaps could support guideline changes and ultimately reduce the burden of HFpEF in this growing patient group.

## Data Availability

No datasets were generated or analysed during the current study.

## Abbreviations

BMI: Body mass index
BSA: Body surface area
BNP: B-type natriuretic peptide
ESC: European Society of Cardiology (ESC)
GIP: Glucose-dependent insulinotropic polypeptide
GLP-1: Glucagon-like peptide-1
H2FPEF: Heavy, hypertension, atrial fibrillation, pulmonary hypertension, elder, and filling pressures
HF: Heart failure
HFA-PEFF: Heart Failure Association Pretest Probability of Heart Failure with Preserved Ejection Fraction
HFpEF: Heart failure with preserved ejection fraction
MBS: Metabolic bariatric surgery
NT-proBNP: N-terminal pro B-type natriuretic peptide
SGLT2: Sodium glucose cotransporter 2

## References

[1] Collaborators GRF (2020) Global burden of 87 risk factors in 204 countries and territories, 1990–2019: a systematic analysis for the global burden of disease study 2019. Lancet 396(10258):1223–1249 33069327 10.1016/S0140-6736(20)30752-2 PMC7566194

[2] Kenchaiah S, Evans JC, Levy D et al (2002) Obesity and the risk of heart failure. N Engl J Med 347(5):305–313 12151467 10.1056/NEJMoa020245

[3] Suthahar N, Meems LMG, Withaar C et al (2022) Relative fat mass, a new index of adiposity, is strongly associated with incident heart failure: data from PREVEND. Sci Rep 12(1):147 34996898 10.1038/s41598-021-02409-6 PMC8741934

[4] Obokata M, Reddy YNV, Pislaru SV et al (2017) Evidence supporting the existence of a distinct obese phenotype of heart failure with preserved ejection fraction. Circulation 136(1):6–19 28381470 10.1161/CIRCULATIONAHA.116.026807 PMC5501170

[5] Ho JE, Lyass A, Lee DS et al (2013) Predictors of new-onset heart failure: differences in preserved versus reduced ejection fraction. Circ Heart Fail 6(2):279–86 23271790 10.1161/CIRCHEARTFAILURE.112.972828 PMC3705220

[6] Tromp J, Claggett BL, Liu J et al (2021) Global differences in heart failure with preserved ejection fraction: the PARAGON-HF Trial. Circ Heart Fail 14(4):e007901 33866828 10.1161/CIRCHEARTFAILURE.120.007901

[7] Chockalingam A (2022) “Obesity-Years” burden may predict reversibility in heart failure with preserved ejection fraction. Front Cardiovasc Med 9:821829 35198616 10.3389/fcvm.2022.821829 PMC8858971

[8] Reddy YNV, Lewis GD, Shah SJ et al (2019) Characterization of the obese phenotype of heart failure with preserved ejection fraction: a RELAX trial ancillary study. Mayo Clin Proc 94(7):1199–1209 31272568 10.1016/j.mayocp.2018.11.037

[9] Iacobellis G, Corradi D, Sharma AM (2005) Epicardial adipose tissue: anatomic, biomolecular and clinical relationships with the heart. Nat Clin Pract Cardiovasc Med 2(10):536–543 16186852 10.1038/ncpcardio0319

[10] Adeva-Andany MM, Domínguez-Montero A, Adeva-Contreras L et al (2024) Body fat distribution contributes to defining the relationship between insulin resistance and obesity in human diseases. Curr Diabetes Rev 20(5):e160823219824 37587805 10.2174/1573399820666230816111624

[11] Zain S, Shamshad T, Kabir A et al (2023) Epicardial adipose tissue and development of atrial fibrillation (AFIB) and heart failure with preserved ejection fraction (HFpEF). Cureus 15(9):e46153 37900360 10.7759/cureus.46153 PMC10612538

[12] Esser N, L’Homme L, De Roover A et al (2013) Obesity phenotype is related to NLRP3 inflammasome activity and immunological profile of visceral adipose tissue. Diabetologia 56(11):2487–2497 24013717 10.1007/s00125-013-3023-9

[13] Porreca E, Di Febbo C, Fusco L et al (2004) Soluble thrombomodulin and vascular adhesion molecule-1 are associated to leptin plasma levels in obese women. Atherosclerosis 172(1):175–180 14709373 10.1016/j.atherosclerosis.2003.09.022

[14] Verboven K, Wouters K, Gaens K et al (2018) Abdominal subcutaneous and visceral adipocyte size, lipolysis and inflammation relate to insulin resistance in male obese humans. Sci Rep 8(1):4677 29549282 10.1038/s41598-018-22962-x PMC5856747

[15] Zeller M, Steg PG, Ravisy J et al (2008) Relation between body mass index, waist circumference, and death after acute myocardial infarction. Circulation 118(5):482–490 18625893 10.1161/CIRCULATIONAHA.107.753483

[16] Bodenstab ML, Varghese RT, Iacobellis G (2024) Cardio-lipotoxicity of epicardial adipose tissue. Biomolecules 14(11) 10.3390/biom14111465 PMC11591820 39595641

[17] Borlaug BA, Jensen MD, Kitzman DW et al (2023) Obesity and heart failure with preserved ejection fraction: new insights and pathophysiological targets. Cardiovasc Res 118(18):3434–3450 35880317 10.1093/cvr/cvac120 PMC10202444

[18] Packer M (2018) Epicardial adipose tissue may mediate deleterious effects of obesity and inflammation on the myocardium. J Am Coll Cardiol 71(20):2360–2372 29773163 10.1016/j.jacc.2018.03.509

[19] Weerts J, Mourmans SGJ, Barandiarán Aizpurua A, et al (2022) The role of systemic microvascular dysfunction in heart failure with preserved ejection fraction. Biomolecules 12(2) 10.3390/biom12020278 PMC8961612 35204779

[20] Wang W, Gao Y, Wang J et al (2024) Prognostic value of epicardial adipose tissue in heart failure with mid-range and preserved ejection fraction: a multicenter study. J Am Heart Assoc 13(24):e036789 39673347 10.1161/JAHA.124.036789 PMC11935535

[21] McDonagh TA, Metra M, Adamo M et al (2023) 2023 focused update of the 2021 ESC guidelines for the diagnosis and treatment of acute and chronic heart failure. Eur Heart J 44(37):3627–3639 37622666 10.1093/eurheartj/ehad195

[22] Marx N, Federici M, Schütt K et al (2023) 2023 ESC Guidelines for the management of cardiovascular disease in patients with diabetes: developed by the task force on the management of cardiovascular disease in patients with diabetes of the European Society of Cardiology (ESC). Eur Heart J 44(39):4043–4140 37622663 10.1093/eurheartj/ehad192

[23] Ndumele CE, Neeland IJ, Tuttle KR et al (2023) A synopsis of the evidence for the science and clinical management of cardiovascular-kidney-metabolic (CKM) syndrome: a scientific statement from the American Heart Association. Circulation 148(20):1636–1664 37807920 10.1161/CIR.0000000000001186

[24] Reddy YNV, Carter RE, Sundaram V et al (2024) An evidence-based screening tool for heart failure with preserved ejection fraction: the HFpEF-ABA score. Nat Med 30(8):2258–2264 38997608 10.1038/s41591-024-03140-1 PMC11570987

[25] Kosyakovsky LB, Liu EE, Wang JK et al (2024) Uncovering unrecognized heart failure with preserved ejection fraction among individuals with obesity and dyspnea. Circ Heart Fail 17(5):e011366 38742409 10.1161/CIRCHEARTFAILURE.123.011366 PMC11214582

[26] Bernhardt V, Babb TG (2016) Exertional dyspnoea in obesity. Eur Respir Rev 25(142):487–495 27903669 10.1183/16000617.0081-2016 PMC9487557

[27] van Dalen BM, Chin JF, Motiram PA et al (2025) Challenges in the diagnosis of heart failure with preserved ejection fraction in individuals with obesity. Cardiovasc Diabetol 24(1):71 39920805 10.1186/s12933-025-02612-z PMC11806779

[28] Todd M (2009) Managing chronic oedema in the morbidly obese patient. Br J Nurs 18(18):1120–4 19966731 10.12968/bjon.2009.18.18.44557

[29] Wearing SC, Hennig EM, Byrne NM et al (2006) Musculoskeletal disorders associated with obesity: a biomechanical perspective. Obes Rev 7(3):239–250 16866972 10.1111/j.1467-789X.2006.00251.x

[30] Daniels LB, Clopton P, Bhalla V et al (2006) How obesity affects the cut-points for B-type natriuretic peptide in the diagnosis of acute heart failure. Results from the Breathing Not Properly Multinational Study. Am Heart J. 151(5):999–1005 16644321 10.1016/j.ahj.2005.10.011

[31] Verbrugge FH, Omote K, Reddy YNV et al (2022) Heart failure with preserved ejection fraction in patients with normal natriuretic peptide levels is associated with increased morbidity and mortality. Eur Heart J 43(20):1941–1951 35139159 10.1093/eurheartj/ehab911 PMC9649913

[32] Obokata M, Kane GC, Reddy YN et al (2017) Role of diastolic stress testing in the evaluation for heart failure with preserved ejection fraction: a simultaneous invasive-echocardiographic study. Circulation 135(9):825–838 28039229 10.1161/CIRCULATIONAHA.116.024822 PMC5330848

[33] Reddy YNV, Tada A, Obokata M et al (2025) Evidence-based application of natriuretic peptides in the evaluation of chronic heart failure with preserved ejection fraction in the ambulatory outpatient setting. Circulation 151(14):976–989 39840432 10.1161/CIRCULATIONAHA.124.072156 PMC12021425

[34] Suthahar N, Meijers WC, Ho JE et al (2018) Sex-specific associations of obesity and N-terminal pro-B-type natriuretic peptide levels in the general population. Eur J Heart Fail 20(8):1205–1214 29855124 10.1002/ejhf.1209 PMC6105520

[35] Reddy YNV, Carter RE, Obokata M et al (2018) A simple, evidence-based approach to help guide diagnosis of heart failure with preserved ejection fraction. Circulation 138(9):861–870 29792299 10.1161/CIRCULATIONAHA.118.034646 PMC6202181

[36] Pieske B, Tschöpe C, de Boer RA et al (2019) How to diagnose heart failure with preserved ejection fraction: the HFA-PEFF diagnostic algorithm: a consensus recommendation from the Heart Failure Association (HFA) of the European Society of Cardiology (ESC). Eur Heart J 40(40):3297–3317 31504452 10.1093/eurheartj/ehz641

[37] Sanders-van Wijk S, Barandiarán Aizpurua A, Brunner-La Rocca HP et al (2021) The HFA-PEFF and H(2) FPEF scores largely disagree in classifying patients with suspected heart failure with preserved ejection fraction. Eur J Heart Fail 23(5):838–840 33012125 10.1002/ejhf.2019 PMC8359393

[38] Selvaraj S, Myhre PL, Vaduganathan M et al (2020) Application of diagnostic algorithms for heart failure with preserved ejection fraction to the community. JACC Heart Fail 8(8):640–653 32535127 10.1016/j.jchf.2020.03.013 PMC8030634

[39] Cox N, Resnic FS, Popma JJ et al (2004) Comparison of the risk of vascular complications associated with femoral and radial access coronary catheterization procedures in obese versus nonobese patients. Am J Cardiol 94(9):1174–1177 15518615 10.1016/j.amjcard.2004.07.088

[40] Ellenberger K, Jeyaprakash P, Sivapathan S et al (2022) The effect of obesity on echocardiographic image quality. Heart Lung Circ 31(2):207–215 34373191 10.1016/j.hlc.2021.06.525

[41] de Simone G, Galderisi M (2014) Allometric normalization of cardiac measures: producing better, but imperfect, accuracy. J Am Soc Echocardiogr 27(12):1275–1278 25479898 10.1016/j.echo.2014.10.006

[42] Aga Y, Acardag Y, Chin JF et al (2024) Improved identification of left atrial enlargement in patients with obesity. Int J Cardiovasc Imaging 40(1):65–72 37882958 10.1007/s10554-023-02981-0 PMC10774171

[43] Jeyaprakash P, Moussad A, Pathan S et al (2021) A systematic review of scaling left atrial size: are alternative indexation methods required for an increasingly obese population? J Am Soc Echocardiogr 34(10):1067-1076.e3 34023453 10.1016/j.echo.2021.05.009

[44] Bermea KC, Lovell JP, Hays AG et al (2024) A machine learning-derived score to effectively identify heart failure with preserved ejection fraction. JACC Adv 3(7):101040 39130016 10.1016/j.jacadv.2024.101040 PMC11312345

[45] Amangurbanova M, Daher R, Asbeutah AA et al (2024) Higher epicardial adipose tissue volume is associated with higher coronary fatty plaque volume and is regulated by waist circumference but not EPA+DHA supplementation. J Clin Lipidol 18(5):e773–e786 39289125 10.1016/j.jacl.2024.06.006

[46] Adamson C, Kondo T, Jhund PS et al (2022) Dapagliflozin for heart failure according to body mass index: the DELIVER trial. Eur Heart J 43(41):4406–4417 36029309 10.1093/eurheartj/ehac481 PMC9622300

[47] Cohen JB, Schrauben SJ, Zhao L et al (2020) Clinical phenogroups in heart failure with preserved ejection fraction: detailed phenotypes, prognosis, and response to spironolactone. JACC Heart Fail 8(3):172–184 31926856 10.1016/j.jchf.2019.09.009 PMC7058514

[48] Courcoulas AP, Belle SH, Neiberg RH et al (2015) Three-year outcomes of bariatric surgery vs lifestyle intervention for type 2 diabetes mellitus treatment: a randomized clinical trial. J Am Med Assoc Surg 150(10):931–940 10.1001/jamasurg.2015.1534 PMC4905566 26132586

[49] Adams TD, Davidson LE, Litwin SE et al (2017) Weight and metabolic outcomes 12 years after gastric bypass. N Engl J Med 377(12):1143–1155 28930514 10.1056/NEJMoa1700459 PMC5737957

[50] Berger S, Meyre P, Blum S et al (2018) Bariatric surgery among patients with heart failure: a systematic review and meta-analysis. Open Heart 5(2):e000910 30613414 10.1136/openhrt-2018-000910 PMC6307626

[51] Miranda WR, Batsis JA, Sarr MG et al (2013) Impact of bariatric surgery on quality of life, functional capacity, and symptoms in patients with heart failure. Obes Surg 23(7):1011–1015 23604694 10.1007/s11695-013-0953-8

[52] Shimada YJ, Tsugawa Y, Brown DFM et al (2016) Bariatric surgery and emergency department visits and hospitalizations for heart failure exacerbation: population-based, self-controlled series. J Am Coll Cardiol 67(8):895–903 26916477 10.1016/j.jacc.2015.12.016

[53] Vest AR, Patel P, Schauer PR et al (2016) Clinical and echocardiographic outcomes after bariatric surgery in obese patients with left ventricular systolic dysfunction. Circ Heart Fail 9(3):e002260 26945045 10.1161/CIRCHEARTFAILURE.115.002260

[54] Leichman JG, Wilson EB, Scarborough T et al (2008) Dramatic reversal of derangements in muscle metabolism and left ventricular function after bariatric surgery. Am J Med 121(11):966–973 18954843 10.1016/j.amjmed.2008.06.033 PMC2604808

[55] Kosiborod MN, Abildstrøm SZ, Borlaug BA et al (2023) Semaglutide in patients with heart failure with preserved ejection fraction and obesity. N Engl J Med 389(12):1069–1084 37622681 10.1056/NEJMoa2306963

[56] Jastreboff AM, Aronne LJ, Ahmad NN et al (2022) Tirzepatide once weekly for the treatment of obesity. N Engl J Med 387(3):205–216 35658024 10.1056/NEJMoa2206038

[57] Kramer CM, Borlaug BA, Zile MR et al (2025) Tirzepatide reduces LV mass and paracardiac adipose tissue in obesity-related heart failure: SUMMIT CMR substudy. J Am Coll Cardiol 85(7):699–706 39566869 10.1016/j.jacc.2024.11.001

[58] Wilding JPH, Batterham RL, Davies M et al (2022) Weight regain and cardiometabolic effects after withdrawal of semaglutide: the STEP 1 trial extension. Diabetes Obes Metab 24(8):1553–1564 35441470 10.1111/dom.14725 PMC9542252

[59] Martins FF, Marinho TS, Cardoso LEM et al (2022) Semaglutide (GLP-1 receptor agonist) stimulates browning on subcutaneous fat adipocytes and mitigates inflammation and endoplasmic reticulum stress in visceral fat adipocytes of obese mice. Cell Biochem Funct 40(8):903–913 36169111 10.1002/cbf.3751

[60] Turkmen Sariyildiz G, Cicek Demir C, Demir ME et al (2023) The evaluation of serum endocan, interleukin-6, and CRP levels following sleeve gastrectomy. Int J Gen Med 16:4737–4744 37877002 10.2147/IJGM.S436213 PMC10591641

[61] Borlaug BA, Zile MR, Kramer CM et al (2025) Effects of tirzepatide on circulatory overload and end-organ damage in heart failure with preserved ejection fraction and obesity: a secondary analysis of the SUMMIT trial. Nat Med 31(2):544–551 39551891 10.1038/s41591-024-03374-z PMC11835708

